# Are They Careful Enough? Testing Consumers’ Perception of Alternative Processing Technologies on the Quality of Organic Food

**DOI:** 10.3390/nu13092922

**Published:** 2021-08-24

**Authors:** Busra Kilic, Emilia Cubero Dudinskaya, Migena Proi, Simona Naspetti, Raffaele Zanoli

**Affiliations:** 1 Department of Agricultural, Food and Environmental Sciences (D3A), Università Politecnica delle Marche, Via Brecce Bianche, 60131 Ancona, Italy; S1087732@univpm.it (B.K.); mproi@agrecon.univpm.it (M.P.); 2 Department of Materials, Environmental Sciences and Urban Planning (SIMAU), Università Politecnica delle Marche, Via Brecce Bianche, 60131 Ancona, Italy

**Keywords:** food processing, packaging, UHT, high-pressure, pulsed-electric fields, pasteurization, micro-wave, consumer research, farm-to-fork, randomized experiment

## Abstract

Given the increasing public interest in how ingredients are processed and the growing demand for organic food products, it is critical to understand consumers’ expectations about the process-related quality of organic products. Consumers perceive organic food to be nutritious, healthy and either natural or less processed, as they are afraid of the loss of nutritional, organoleptic and sensory properties of the food products. However, alternative food processing technologies might generate healthy and safe food options with nutritional quality properties. Simplified communication schemes might help to overcome this barrier for the consumer. The main objective of this study is to propose a working definition of “careful processing” for organic products and test its consistency through an experiment, while being used to rate different processing methods by consumers. Results show that the proposed definition allows the consumer to consistently rate alternative processing technologies. Consumers tend to score alternative processing technologies such as pulsed electric fields and microwaves as less careful, supporting the idea that organic consumers want as little man-made interference in their food products as possible. Results show that a simple but effective definition of careful processing may help consumers to distinguish more organic food products from conventional ones, no matter which communication scheme is used.

## 1. Introduction

Organic food systems involve different actors. Therefore, to provide high-quality diets with higher nutritional content, policymakers need to ensure that all stages of these systems work in harmony. Therefore, all processes and activities involved in food production (e.g., processing, storage, etc) should be considered carefully [1]. However, process-related characteristics have been overlooked to date in the organic food process [2]. There is still a lack of mandatory standards and indications specifically related to organic food processing [3]. This is an important issue to address, as urbanization and changes in lifestyle raise the demand for processed foods that are easily transportable and storable [4,5,6].

Food processing technologies have ensured food safety. They have provided many benefits to both consumers and producers, such as extending the shelf life of unprocessed foods, enabling their storage for more prolonged use and making them edible and more convenient [7,8]. Although both thermal and non-thermal processing technologies can be used to process foods, the food industry has been applying heat treatments to pasteurize or sterilize food for many years [9]. This has been done mainly because of the perceived benefits of pasteurization in terms of efficacy and the safety of the end-products [10,11,12]. However, previous studies showed that different processing technologies could have diverse effects on different dimensions of food quality, including changes in the products’ sensory, biochemical and nutritional characteristics [13,14,15].

For organic consumers, the nutritional and sensory quality of organic processed food is dependent on a range of factors from farm to fork, including the processing technologies. In particular, processing technologies with minimal or no changes in foods’ nutritional and sensory properties could offer attractive products with fresh-like taste to consumers [11,16,17]. Obtaining more “natural”, high quality, and safe products with a high nutritional value has led the food industry to pursue intensive research on alternative food processing and packing technologies to achieve higher-quality and safer foods in more efficient ways. As a result, the food industry has recently used alternative food processing technologies [13,14,15,18] such as high-pressure processing (HPP), pulsed electric fields (PEF) and microwave processing. Among the packaging methods, the industry has developed modified atmosphere packaging (MAP), edible-film coating and active packaging [13,14,19,20]. In general, these methods have all been identified to reduce processing time and temperature while minimizing quality losses and input energy during production [13,14,18,19,20,21]. Therefore, their effective implementation may meet the needs of potential organic consumers who want safe, tasty and easy-to-use organic processed products with more “natural” characteristics, fresh-like taste and long shelf-life [22].

Most consumers perceive organic food as a product that has been processed naturally and with health-related benefits [23]. Although much work has been done on consumer perception and the use of alternative food processing methods [9,24,25], very little is known about organic consumers’ perception of food processing methods in organic food production. Naspetti and Zanoli [26] demonstrated that current organic consumers seem unaware of organic food production and processing methods. Organic consumers demand more information. They also desire to distinguish organic products from conventional ones in terms of the processing methods applied. However, as these process-related attributes cannot be judged through experience, they become a question of credible information [27]. Better communication seems fundamental to organic consumers’ awareness.

Informing organic consumers about processing methods in a simplified way might be a good alternative that would also decrease their cognitive effort. Organic consumers expect that organic food is processed with “care” [28], given that “care” is a principle and an essential value in organic food production [19]. New technologies should be evaluated on how they fulfil “careful processing” in the organic industry, offering consumers an understandable tool when evaluating organic products. However, there is still no consent on what “care” means in organic food processing. 

Previous research on the topic of this study has been limited. There is still confusion in the organic industry regarding a clear definition for careful processing. Various authors [3,29,30] proposed definitions of careful processing focused on the product, considering the maximization in the presence of essential elements while avoiding undesirable compounds or nutritional losses. However, Kahl et al. [3] also highlighted the need for a broader definition that links carefulness to three key dimensions: the product, the environment and the people. 

This study aimed to test if the concept of “careful processing” could be used to consistently rate different processing methods for organic food while controlling for different “carefulness” communication schemes through a randomized experiment.

A working definition for “careful processing” was developed with the help of a panel of experts, based on previous research [3,30] and with the principles of organic production [31]. These include the three dimensions of “care” related to food quality, environment, and human health [29]: 

“Careful processing refers to methods that aim to:

(a) preserve the nutritional and sensory quality of raw materials from organic farming by limiting the use of additives,
(b) minimize the risks for consumer and worker health while promoting fair supply-chains, and
(c) limit the impact on the environment by:
  - reducing the use of water and energy,
  - optimizing waste management, and
  - promoting recyclable/reusable packaging.”

In this study, the validity of this definition was tested by scoring different alternative processing methods. We expected that:

**Hypothesis 1 (H1).**  *The careful processing definition allows to rate consistently different processing methods.*

Previous studies have established that front-of-pack labelling, such as traffic light nutrition labels (green, red, and amber), are a helpful tool to communicate with consumers about making healthier choices [32,33,34]. Zhang et al. [35] confirmed that the traffic-light inspired labels (green, red, and amber) may be a more effective means by which to communicate to consumers than purely numeric guideline-daily-amounts labels. Some studies have pointed out that the color-coded scheme is easier to interpret by the consumer [36]. However, others have found that a monochromatic scheme is more effective than a color-coded scheme in capturing consumer attention faster [37,38]. 

Given this ambiguity, and the fact that a scoring task is not as passive as viewing a label, we expected that:

**Hypothesis 2 (H2).**  *The type of communication scheme (mono-chrome vs. color-coded) does not significantly influence how the technologies are rated.*

A mixed factorial randomized experiment was designed to test these hypotheses.

## 2. Materials and Methods

### 2.1. Participants

A total of 130 organic consumers, older than 18 years old, potentially representative of the population [39] and largely suitable for experimental work [40,41,42] were recruited from Amazon’s Mechanical Turk. Experiments require far fewer subjects than methods such as surveys [39]. Following the recommendation of [39] we have used the software G*Power version 3.1. (Heinrich Heine University, Düsseldorf, Germany) [43] to calculate our sample size. Keeping a power of 0.8, which is the minimal accepted [39,44], and a small effect size of 0.12, results show that the total sample size should be 130 respondents, which is exactly our sample size.

Of the respondents, 115 participants (88%) were occasional organic consumers, reporting a certified organic food products consumption between 5% to 50%. The remaining 15 participants (12%) were regular organic consumers, implying that more than 50% of the food products they buy are certified organic.

Participants with a red-green color deficiency were excluded from the experiment, using the simplified 6 plates (plate numbers; 1, 2, 4, 8, 10, 14) version of the highly reliable Ishihara color test [45]. This was a crucial step to avoid any bias, as the study included color scales. Participants were also informed that the information they will provide will considerably contribute to new scientific knowledge on organic food processing methods and may benefit them as organic consumers.

### 2.2. Experimental Design

The study consisted of a classification task of 8 processing methods (thermal: pasteurization (control), UHT, microwave; non-thermal: modified atmosphere, pulsed electric fields, high-pressure preservation, edible coating, active packaging) measured with 2 communication schemes for “careful processing” (monochromatic bar scale vs. color bar scale) and mixed factorial design to check that the type of communication scheme does not influence the classification of the food processing technology. 

The eight processing technologies were chosen according to their current and potential application in the food industry [13,14,19,20]. Food processing technologies which are familiar (pasteurization processing) and less-familiar (alternative food processing technologies) to consumers were included in the study [46,47].

### 2.3. Procedure

Conveying unbiased scientific and technological information to ordinary consumers might be difficult [48,49]. Therefore, after being shown the definition of “careful processing”, participants were shown a short (3 min) cartoon video (Link for the informative video which was created for the present research: https://youtu.be/Veks_qH_OcM) (accessed on 3 August 2020) with textual information on the eight food processing technologies. The video presented a short, neutral and unbiased description of each processing technology (See Appendix A for details). Pasteurization was presented as the current standard thermal technology while the other as thermal/non-thermal alternatives. The video was designed with short and plain-language definitions to enhance the understandability of complex technologies. The specific benefits or risks of each technology were not discussed to avoid generating bias in the subjects. 

The participants’ attention to the video was tested through a control multiple-choice question regarding the information presented in the video. The respondents were asked to select from a list of food processing technologies which methods had not been presented in the video. Only those participants who responded correctly the control question about the video were allowed to continue the experiment. In this way, it was possible to ensure that the participants were involved and attentive to the video content. 

**Figure 1:**
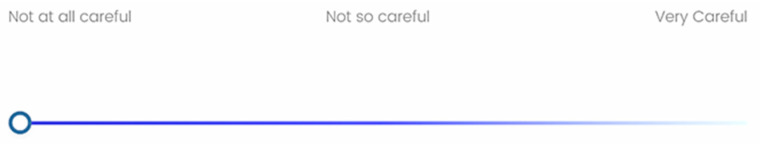
The monochromatic communication scheme.

**Figure 2:**
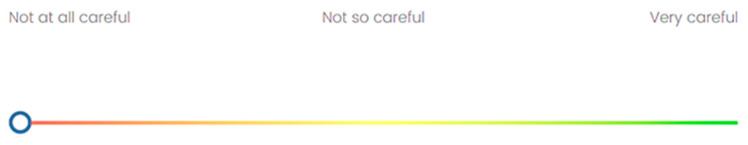
The multi-color communication scheme.

Participants were randomly assigned to one of two communication schemes (experimental conditions). The first communication scheme consisted of a monochromatic color bar scale (dark blue = not at all careful, blue = not so careful, whitish pale blue = very careful) presented in Figure 1. The second one included a multi-color bar scale (red = not at all careful, yellow = not so careful, green = very careful.) shown in Figure 2.

### 2.4. Measures

The dependent variable (DV) consisted in a continuous scale measuring “carefulness” (as from the definition) ranging from 0 to 100 (0 = Not at all careful, 100 = Very careful) for each of the eight food processing technologies.

The independent variable consisted of the treatment measured using the two different communication schemes (monochromatic and color). An interaction of method-by-scheme was also taken into consideration in the study. 

### 2.5. Statistical Analysis

Using the software Stata/MP version 17 (StataCorp, College Station, TX, USA), a one between-factor repeated measures ANOVA was run on the sample to determine if there were differences in carefulness ratings of the different food processing technologies due to the communication scheme used. Moreover, pairwise-comparisons of predictive margins were also calculated for each scheme (monochromatic and color).

## 3. Results

The results showed that the communication scheme did not elicit statistically significant differences in mean carefulness score, F(1, 128) = 1.91, *p* = 0.17. Consistent with hypothesis H2, the method-by-scheme interaction was also not statistically significant (F(7, 896) = 1.13, *p* = 0.34).

However, there was a statistically significant effect of the method on carefulness ratings, F(7, 896) = 13.50, *p* < 0.001. 

Pooling the error allows the examination of the carefulness score for each technology in each scheme and estimate simple effects, using pasteurization as the reference method. In both schemes, all methods were rated not significantly different in carefulness compared to pasteurization, except for alternative technologies (microwave and pulsed electric fields), which were considered less careful in all schemes (Table 1).

Pairwise comparisons (detailed results reported in Appendix B) show that packaging methods generally are perceived as more careful than processing methods. 

Such results indicate that the proposed definition allows to consistently rate the processing technologies under study, not falsifying Hypothesis H1.

## 4. Discussion

Consumers tend to exhibit mixed attitudes towards organic processed food with ‘traditional’ organic consumers tending to have a negative image of processed food [50]. Consumers perceive a food-health imbalance among processed food products, especially regarding their nutrition interface and its relevance within diet-health debates [8,51]. Although some studies have shown that highly processed food products might have negative consequences on human health [52], these results depend on the kind of food processing. 

Organic consumers expect their products to be processed with “care” [28], while health is the main motivation for organic food consumption [22,53,54]. Health-conscious consumers are expected to prefer ‘minimally’ processed food, since they are expected to preserve the nutritional quality of food [55]. Besides, consumers who are health conscious tend to prefer visuals of unprocessed foods on packaging, since they symbolize naturalness [56].

Our study evaluated organic consumers’ perspectives about food processing technologies (eight processing and packaging methods) according to a “careful processing” definition, which was presented to respondents through different communication schemes. No significant differences were found between the two communication schemes (monochromatic vs. polychromatic).

However, differences were found in how participants scored the level of carefulness of the various processing technologies. In general, packaging methods (MAP, active packaging, edible coating) were evaluated as “careful” by the participants and perceived alike regardless of their characteristics. This can be explained by the fact that organic consumers give substantial weight to the perceived naturalness of the product. Packaging methods require minimal human intervention over the properties of the food product, given consumers a sense of an “unprocessed” and “natural” product [57,58]. Among processing methods, pasteurization is one of the oldest and most widely used in the food industry [12], which makes it quite familiar to consumers, given that they have had enough time to appreciate and experience the benefits of this processing technology [59]. Previous research has shown that familiarity and trust affect the organic consumers’ perception much more than the tangible benefits of the processing methods [60,61].

No statistically significant difference in terms of carefulness was found between pasteurization and all the other methods except for microwave and PEF. Microwave processing was considered the least careful method according to the “careful processing definition”. An explanation for this result can be the fact that the primary motivation of organic consumers is health, which is commonly linked to “naturalness” and “purity” [54]. Given the limited knowledge that organic consumers have regarding food processing technologies, they associate organic processing with food produced naturally, home-grown food and in an environmentally friendly way [23]. As a consequence, any technology which, in the consumers’ mind, might affect the “purity” or “naturalness” [58,62] of the organic product will be perceived as not in harmony with the nature of organic production and will be rejected by organic consumers. Another possible explanation could be general skepticism about new food technologies, regardless of how these processes affect various dimensions of organic food quality [9,63]. Communication, transparency and a trusted knowledge source are paramount in shaping consumer perceptions regarding organic processed food [63,64].

Likewise, consumers might perceive alternative technologies applied to food processing as risky and unfamiliar [65,66]. The unfamiliarity and uncertainty linked to introducing new technologies might lead organic consumers to resist such innovations, perceiving themselves as victims rather than beneficiaries [67]. These results show that the skeptical attitude toward new food processing technologies is caused mainly by a knowledge deficiency, which fits with earlier findings [68,69]. In general, trust in the (natural) food supply chain positively influences purchase intention, especially when consumers lack familiarity and knowledge of a specific food category [70].

Therefore, accurate and simplified information could minimize consumers’ concerns and improve their acceptance and consumption of food produced with alternative methods. However, as Fischer and Frewer [59] stated, risks and benefits are related constructs that are not independently evaluated by consumers [60]. Therefore, in food which is perceived to be unfamiliar, the information describing the benefits may only influence consumers if this information is presented before the information outlining the risks.

## 5. Conclusions

In this study, a working definition of careful organic processing was provided and communicated to consumers. We provided evidence that this definition allows consumers to consistently distinguish and rate alternative processing and packaging methods, notwithstanding how carefulness is communicated. Results are relevant for the debate initiated by Kahl et al. [3] on the ‘starting’ definitions of organic food processing.

The working definition proposed and tested in this study encompasses both the definitions of ‘minimal’ and ‘careful’ processing [71] which are usually referred to when defining organic food processing. The definition also broadly encompasses the concept of ‘food naturalness’ [62,72], which has been recently defined and tested. We believe the definition may be useful to further develop an operational, multi-dimensional approach to organic food processing, aiming to: (1) limit the impact of processing on the nutritional and sensory qualities of organic food, while (2) enhancing shelf life and (3) taking care of people and any biotic and abiotic factors both directly and indirectly involved in the processing. 

Future research could investigate if communicating the level of carefulness of processed organic food could create value for organic consumers [23]. Since communicating carefulness may be associated with different risk perceptions, the role of associated consumer emotions should also be addressed in future research.

## Figures and Tables

**Table 1:** Simple effects of food processing technologies at each communication scheme.

Method/Scheme	Contrast	Std. Err.	t	P > t
(active vs. past) Mono	−1.692308	4.247585	−0.40	0.690
(active vs. past) Color	−3.661538	4.247585	−0.86	0.389
(edible vs. past) Mono	−1.630769	4.247585	−0.38	0.701
(edible vs. past) Color	2.169231	4.247585	0.51	0.610
(hpp vs. past) Mono	−4.769231	4.247585	−1.12	0.262
(hpp vs. past) Color	0.6153846	4.247585	0.14	0.885
(map vs. past) Mono	1.492308	4.247585	0.35	0.725
(map vs. past) Color	−1.169231	4.247585	−0.28	0.783
(micro vs. past) Mono	−15.92308	4.247585	−3.75	0.000
(micro vs. past) Color	−21.67692	4.247585	−5.10	0.000
(pef vs. past) Mono	−11.58462	4.247585	−2.73	0.006
(pef vs. past) Color	−9.723077	4.247585	−2.29	0.022
(uht vs. past) Mono	−2.830769	4.247585	−0.67	0.505
(uht vs. past) Color	−6.861538	4.247585	−1.62	0.107

Legend: past = pasteurization (reference); active = active packaging; edible = edible coating; hpp = high pressure processing; map = modified atmosphere; micro = microwave; uht = ultra-high temperature.

## Data Availability

Not applicable.

## Appendix A

**Table A1:** Statements used in informative video.

Method	Definition
Pasteurization	Uses the heating effect (below 100 °C) for preserving food products
Ultra-high-temperature processing	Uses a high temperature (not less than 135 °C)/short time for preserving food products
High-pressure processing	Uses the pressure at room temperature for preserving food products
Pulsed electric field processing	Uses short electric pulses for preserving food products
Microwave processing	Uses the microwave energy to generate heating for preserving food products
Active packaging	Uses the packaging material inside of the packaging for preserving packaged food products
Modified atmosphere packaging	Uses the protective atmosphere inside of the packaging for preserving packaged food products
Edible-film coating	Uses thin layers of edible materials for preserving the food products

## Appendix B

**Table A2:** Pairwise-comparisons of predictive margins for monochromatic color scheme.

	Delta-Method		Unadjusted	
Method	Contrast	Std. Err.	t	P > t
edible vs. active	0.0615385	3.654901	0.02	0.987
hpp vs. active	−3.076923	3.654901	−0.84	0.4
map vs. active	3.184615	3.654901	0.87	0.384
micro vs. active	−14.23077	3.654901	−3.89	0
past vs. active	1.692308	3.654901	0.46	0.643
pef vs. active	−9.892308	3.654901	−2.71	0.007
uht vs. active	−1.138462	3.654901	−0.31	0.756
hpp vs. edible	−3.138462	3.654901	−0.86	0.391
map vs. edible	3.123077	3.654901	0.85	0.393
micro vs. edible	−14.29231	3.654901	−3.91	0
past vs. edible	1.630769	3.654901	0.45	0.656
pef vs. edible	−9.953846	3.654901	−2.72	0.007
uht vs. edible	−1.2	3.654901	−0.33	0.743
map vs. hpp	6.261538	3.654901	1.71	0.087
micro vs. hpp	−11.15385	3.654901	−3.05	0.002
past vs. hpp	4.769231	3.654901	1.3	0.192
pef vs. hpp	−6.815385	3.654901	−1.86	0.063
uht vs. hpp	1.938462	3.654901	0.53	0.596
micro vs. map	−17.41538	3.654901	−4.76	0
past vs. map	−1.492308	3.654901	−0.41	0.683
pef vs. map	−13.07692	3.654901	−3.58	0
uht vs. map	−4.323077	3.654901	−1.18	0.237
past vs. micro	15.92308	3.654901	4.36	0
pef vs. micro	4.338462	3.654901	1.19	0.236
uht vs. micro	13.09231	3.654901	3.58	0
pef vs. past	−11.58462	3.654901	−3.17	0.002
uht vs. past	−2.830769	3.654901	−0.77	0.439
uht vs. pef	8.753846	3.654901	2.4	0.017

**Table A3:** Pairwise-comparisons of predictive margins for color-coded scheme.

	Delta-Method		Unadjusted	
Method	Contrast	std. err.	t	P > t
edible vs. active	5.830769	3.654901	1.6	0.111
hpp vs. active	4.276923	3.654901	1.17	0.242
map vs. active	2.492308	3.654901	0.68	0.495
micro vs. active	−18.01538	3.654901	−4.93	0
past vs. active	3.661538	3.654901	1	0.317
pef vs. active	−6.061538	3.654901	−1.66	0.098
uht vs. active	−3.2	3.654901	−0.88	0.382
hpp vs. edible	−1.553846	3.654901	−0.43	0.671
map vs. edible	−3.338462	3.654901	−0.91	0.361
micro vs. edible	−23.84615	3.654901	−6.52	0
past vs. edible	−2.169231	3.654901	−0.59	0.553
pef vs. edible	−11.89231	3.654901	−3.25	0.001
uht vs. edible	−9.030769	3.654901	−2.47	0.014
map vs. hpp	−1.784615	3.654901	−0.49	0.625
micro vs. hpp	−22.29231	3.654901	−6.1	0
past vs. hpp	−0.6153846	3.654901	−0.17	0.866
pef vs. hpp	−10.33846	3.654901	−2.83	0.005
uht vs. hpp	−7.476923	3.654901	−2.05	0.041
micro vs. map	−20.50769	3.654901	−5.61	0
past vs. map	1.169231	3.654901	0.32	0.749
pef vs. map	−8.553846	3.654901	−2.34	0.019
uht vs. map	−5.692308	3.654901	−1.56	0.12
past vs. micro	21.67692	3.654901	5.93	0
pef vs. micro	11.95385	3.654901	3.27	0.001
uht vs. micro	14.81538	3.654901	4.05	0
pef vs. past	−9.723077	3.654901	−2.66	0.008
uht vs. past	−2.830769	3.654901	−0.77	0.439
uht vs. pef	8.753846	3.654901	2.4	0.017
